# Psychological Interventions for PTSD Children and Adolescents Within Two Years of Natural Disasters: A Systematic Scoping Review

**DOI:** 10.1007/s40653-026-00847-w

**Published:** 2026-03-17

**Authors:** Qi Yue Chen, See Heng Yim, Andrea Pickering

**Affiliations:** 1 University College London, London, UK; 2 University of Hong Kong, Hong Kong, China; 3 Canterbury Christ Church University, Canterbury, UK

**Keywords:** PTSD, Natural disaster, Children and adolescent, Systematic review, MHPSS

## Abstract

Natural disasters are becoming more common in response to climate change. There is growing recognition of the traumatic impact on the psychological development of children and young people (CYP), due to their vulnerability. Considering the high prevalence of PTSD among natural disaster survivors, it is crucial to identify and implement effective psychological interventions for CYP, particularly in the early post-traumatic period to mitigate worsening implications for mental health. This systematic scoping review aims to summarize the current types of psychological and psychosocial interventions for CYP’s PTSD within two-year post-disaster, settings that they were used in, as well as their effectiveness. This review adhered to PRISMA-ScR and TIDieR checklist to screen, evaluate, extract and synthesise studies. Inclusion criteria included CYP who were suffering from stress-related symptoms post-disaster. A total of 18 papers showing 16 unique studies were included, interventions included CBT, Narrative Exposure Therapy (NET), psychological first aid (PFA), play therapy and integrative programmes. Most were conducted in school contexts. There was a mixture of task-sharing interventions (delivered by non-specialists) and psychologist-delivered interventions. Most studies found significant reduction in PTSD symptoms except for a study on play therapy and another on school-based teacher-delivered psychosocial programme. Psychological interventions were effective in treating CYP’s PTSD within two years after a disaster. However, this area is still under-researched and critical evidence is still lacking, such as the comparison of treatments and optimal timing of interventions, longitudinal evaluations, as well as how mitigating factors affect their effectiveness.

**Supplementary Information:**

The online version contains supplementary material available at https://doi.org/10.1007/s40653-026-00847-w.

## Introduction

Natural disasters are catastrophic events caused by natural phenomena, leading to large-scale casualties and environmental destruction (Metych, 2024), including hurricanes, earthquakes, tsunamis, storms, and floods. Factors such as urbanization, population growth and climate change have exacerbated the intensity, frequency and geographic scope of natural disasters (Tin et al., 2024). These are expected to escalate further by the end of this century (Hahn et al., 2022). Over the past three decades, an average of 130 million people have been affected annually, with more than 40,000 deaths (Song et al., 2023). In addition to physical injuries, casualties and economic damage, natural disasters can lead to psychological distress and long-term mental health conditions, including post-traumatic stress disorder (PTSD) (Carmassi et al., 2020), anxiety, depression, and suicidality (Patel et al., 2020). These are major challenges for public health and social development (Stanke et al., 2012).

Children and young people (CYP, population below or at 18 years of age) are particularly vulnerable to disaster trauma. Due to the range in developmental stages, reduced coping skills, and cognitive and emotional immaturity, the psychological responses in CYP differ from those in an adult population and are more difficult to discern (Kar, 2009). As a result, parents, teachers, and even mental health professionals may underestimate the severity and long-term impact of their stress (Cohen, 2010). Each year, an estimated 175 million children are affected by climate change-related natural disasters (Midtbust et al., 2018), disrupting fundamental adaptive systems for their development (Masten & Obradovic, 2008). Additionally, secondary disaster stressors, including displacement, separation from loved ones, lack of basic resources, disrupted education, and reduced social support, can further exacerbate their psychological burden (Le Roux & Cobham, 2022; Powell et al., 2021).

Acute Stress Disorder (ASD), occurring within three days to a month post-trauma (DSM-5, 2013), is more typically characterised by psychological hyperarousal, re-experiencing, and sleep problems (Kar, 2009). The pooled prevalence of ASD was 16.5% (95% CI 10.6–23.4%) (Walker et al., 2020). PTSD is the most identified mental disorder in CYP who have been exposed to natural disasters (Le Roux & Cobham, 2022), with prevalence rates ranging from 5% to 43% (Kar et al., 2007). In addition, exposure to natural disasters also leads to both internalising (such as depression, anxiety) and externalising problems beyond PTSD, although the effect sizes for externalising problems tend to be smaller (Rubens et al., 2018). CYP meeting PTSD criteria may also experience co-occurring depression and anxiety following natural disasters such as a hurricane (La Greca et al., 2017). Taken together, exposure to trauma during childhood or adolescence can severely impact CYP’s emotional, cognitive, and social development, leading to poor educational and mental health outcomes, as well as difficulties in learning, memory, emotional regulation, and social relationships (Magruder et al., 2017).

According to Scheeringa et al. (2011), PTSD symptoms vary across developmental levels because diagnostic criteria were originally developed for adults and did not account for the cognitive, verbal, and emotional maturity of younger children. While adults typically report fear, helplessness, or horror, preschoolers exhibit a broader range of emotions, including sadness, confusion, crying, anger, and being unusually quiet; irritability may be expressed through temper tantrums. Regarding the re-experiencing symptoms, rather than verbally expressing intrusions, pre-pubertal children may express trauma through play or reenactment in their games, drawings, or verbal expressions. In contrast, adolescents possess cognitive and verbal capacities to express their symptoms, such as reporting re-experiencing symptoms and persistent negative emotional states. Compared to both children and adults, they are also more prone to aggressive and impulsive behaviours (Hamblen & Barnett, 2016). Despite the high vulnerability to PTSD in young children, PTSD symptoms can be difficult to detect in this population, as they may struggle to articulate their feelings and cognitions in self-report (Danzi et al., 2024; U.S. Department of Veterans Affairs, 2025).

Given the high prevalence of PTSD among natural disaster survivors, there is an urgent need to identify and implement effective interventions to mitigate the impact of PTSD on CYP. Longitudinal studies have identified four trauma trajectories: chronic (persistent high symptoms), recovery (initial symptoms that decrease over time), moderate-stable (moderate symptoms that remain relatively constant), and low-decreasing or stable-low (minimal or no symptoms) (Lai et al., 2021). The largest proportion falls into the resilient and recovery groups; fewer people experience chronic trauma. Older youth tend to have lower odds of chronic distress (Lai et al., 2021). Higher trauma exposure, additional life stressors, less social support, and negative coping strategies are associated with less favourable trajectories (Witt et al., 2024). This highlights the importance of tailoring interventions to those with different levels of needs and considering individual and community-level factors in promoting resilience and recovery. Therefore, a stepped care approach is recommended for post-disaster psychosocial recovery, which includes three levels (Gibbs et al., 2021). Level 1 (Universal Care) provides support, education, and self-care strategies such as relaxation techniques and social connections, including Psychological First Aid (PFA). Level 2 (Selective & Indicated Interventions) targets individuals with mild or subclinical symptoms, offering skill training to enhance coping and reduce PTSD risk, with interventions like Skills for Psychological Recovery and Skills for Life Adjustment and Resilience. Level 3 (high-intensity, evidence-based psychological therapies) is for individuals diagnosed with PTSD or other trauma-related disorders and requires treatment by specialised mental health professionals, including Cognitive Behavioural Therapy (CBT), Eye Movement Desensitisation and Reprocessing (EMDR), and other targeted treatments (Gibbs et al., 2021). Another well-known stepped care framework is the Inter-Agency Standing Committee (IASC) pyramid, which includes interventions ranging from community cultural activities to specialised psychological interventions (IASC, 2007).

There is a multitude of literature describing and evaluating the effectiveness of psychological interventions after disasters such as earthquakes, tsunamis, and hurricanes, as well as their implementation. Newman et al. (2014) also found in their meta-analytic review that CYP who received psychological interventions were significantly better in PTSD symptoms than those in control or waiting list groups. Similarly, Brown et al. (2017) concluded through a meta-analysis and systematic review that CBT, Narrative Exposure Therapy (NET), EMDR, and classroom-based interventions showed similar effects on PTSD occurring after man-made or natural disasters, and that the higher the level of therapist training, the better the effect. However, Le Roux and Cobham (2022) reported that CYP continued to have symptoms after the intervention, but research was inconsistent in terms of when and how those symptoms were measured.

Although literature exists regarding the intervention effectiveness, prior research has been inconsistent regarding when and how the symptoms were measured (Le Roux & Cobham, 2022). Also, to our knowledge, a systematic review of interventions specifically for acute or early PTSD for CYP following natural disasters has not been completed. Early interventions for PTSD aim to reduce the impact of secondary stressors, which may be a critical or sensitive phase affecting their future development (Shalev, 2003). In addition, according to Newman et al. (2014), interventions that took place within four months after the disaster produced the largest impacts, supporting the need for early interventions. Therefore, understanding which intervention is most effective in the first two years post-disaster is critical to improving survivors’ outcomes and psychological conditions.

Given the emerging nature of this field, the diversity of intervention types, and the lack of previous synthesis focusing on the early post-disaster period, a scoping review is well-suited to identify knowledge gaps, explore key characteristics of interventions, and clarify how research has been conducted (Munn et al., 2018), thus laying the groundwork for future systematic reviews or practice recommendations.

This review aimed to summarise and synthesise different types of interventions that are used for PTSD in CYP within two years post-disaster, the implementation setting, the outcome/effectiveness, as well as the limitations of previous studies. This review mainly focused on stress reactions (e.g., PTSD and ASD) rather than other types of mental disorders, due to their high prevalence and severity. PTSD in CYP is often severely disabling, with a wide range of symptoms that affect functioning across multiple domains (Kowalik et al., 2011). Thus, targeting PTSD as a core treatment goal allows for well-established and evidence-based interventions to support an individual’s recovery as well as to improve symptoms of other mental illnesses, such as anxiety, depression, and disruptive behaviours (Cobham & McDermott, 2024).

The review sought to answer the following questions:

1. What contexts and settings were these interventions implemented in?
2. What types of child-focused psychosocial interventions targeting stress reactions were used during the first two years after a natural disaster?
3. How effective were these interventions in achieving their intended outcomes?

## Method

### Search Terms and Selection Criteria

This scoping review followed the guidelines outlined in the Preferred Reporting Items for Systematic Reviews and Meta-Analysis extension for Scoping Reviews (PRISMA-ScR; Page et al., 2021). Five databases (PsycINFO, PubMed, EMBASE, MEDLINE and SCOPUS) were searched in November 2024. The final search strategies for all the databases are presented in Appendix 1.

Qualitative, quantitative and mixed-methods studies were all included in the search to take into account the diversity of approaches currently used for the treatment of acute and early trauma in CYP following natural disasters. Inclusion criteria included.

- Psychological or psychosocial interventions delivered within 2 years following the occurrence of a natural disaster.
- CYP (population below or at 18 years of age) were directly exposed to natural disasters.
- Published after year 2000 and published in English, peer-reviewed journals.
- Interventions focused on PTSD, ASD or Post-Traumatic Stress Symptoms (PTSS).
- Studies used psychometric scales to assess the effectiveness of interventions.

Studies were excluded if:

- Interventions took place over two years after the occurrence of natural disasters.
- They were book chapters, reviews, dissertations, and conference proceedings.

### Critical Appraisal and Data Extraction

The Mixed Methods Appraisal Tool (MMAT; Hong et al., 2018) was used to identify the quality of the studies. For each question, if the answer met the quality criteria, 1 point would be given; if the answer was negative, 0 point would be given; and if the answer was “can’t tell”, which means the study did not provide with appropriate information or the answer was partly agree, 0.5 point would be given. Due to limited empirical research in this area and because the current study was a scoping review, the quality appraisal did not prohibit the inclusion of studies. However, their relative quality will be taken into account when analyzing their findings and evaluating the strength of evidence.

Following the critical appraisal, data were extracted according to the following categories, which were study design, settings (countries, disaster types, populations, places and intervention starting time), measurements, interventions (types, forms and qualifications of therapists), and study outcomes. In addition, the Template for Intervention Description and Replication (TIDieR) checklist (Hoffmann et al., 2014) was used to help report details of intervention implementation. If the relevant information was not provided in the original studies, N/A was marked respectively.

A narrative synthesis approach was adopted to analyse and summarise the extracted data. This process involved the following steps: key findings and study characteristics were first summarised in tables and grouped based on their settings and intervention types; the effectiveness of the interventions was then compared across studies, followed by a critical reflection and synthesis of the robustness of the study findings.

### Studies Selection

**Fig. 1:**
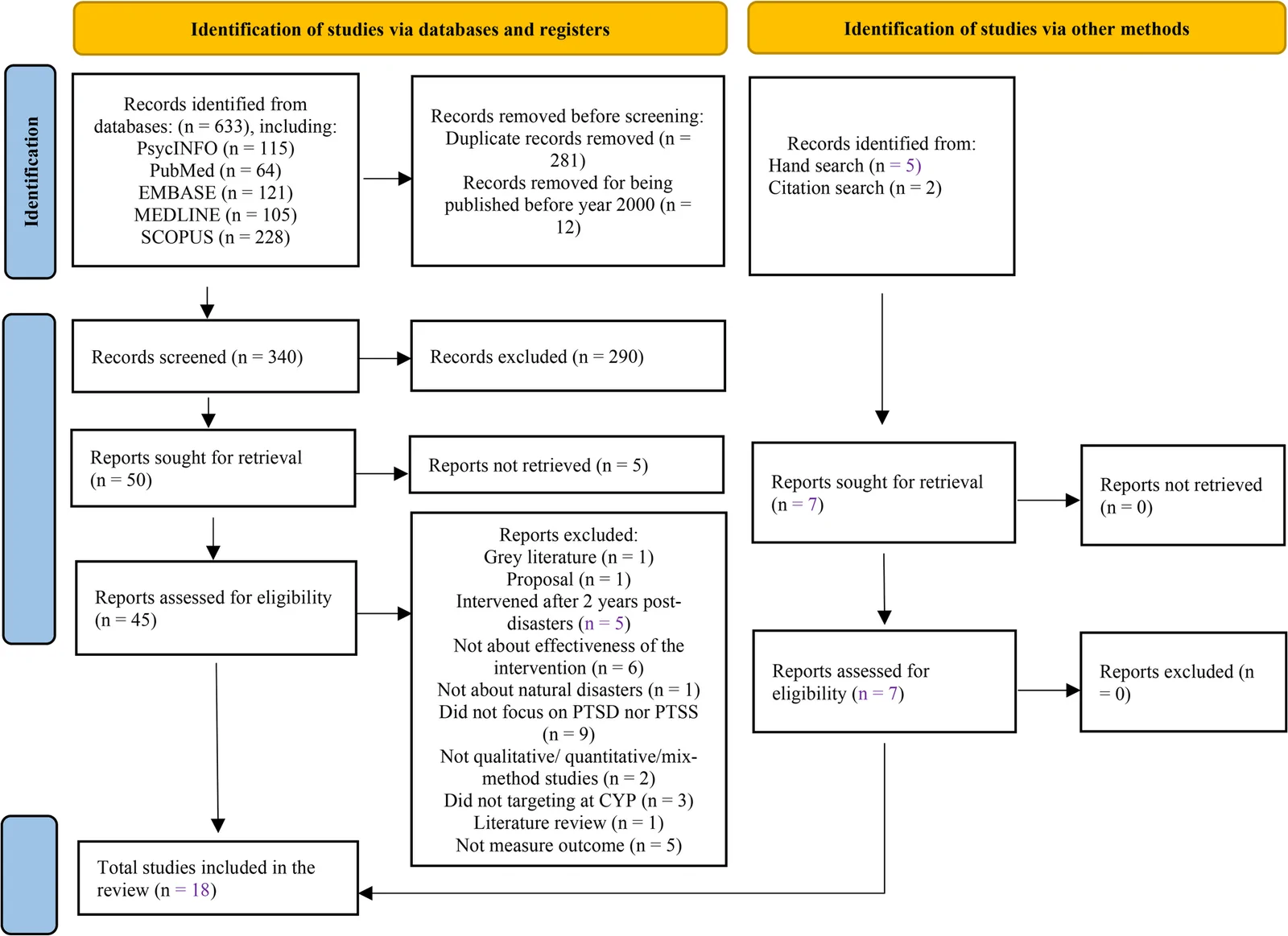
PRISMA-ScR Diagram

A total of 633 studies were imported for screening. Two hundred sixty-two duplicates were removed automatically by EndNote, and 19 were removed manually. Figure 1 presents the PRISMA-ScR (Page et al., 2021) flow diagram, which outlines the details of the research screening process and provides reasons for the exclusions.

QYC and SHY independently screened the studies. The two reviewers met to discuss and resolve any inconsistencies in screening or inclusion decisions through consensus.

## Results

Overall, a total of 18 papers were included in this scoping review, with 10 being randomised controlled trials (RCTs). In addition, 2 of the 18 studies (Goenjian et al., 2021; Wolmer et al., 2005) were follow-up studies of Goenjian et al. (2005) and Wolmer et al. (2003), meaning that a total of 16 unique empirical studies were included.

### Study Quality

The results of critical appraisal are shown in Appendix 2. Among the included studies, the majority scored above 5, indicating moderate to high methodological quality. Only one study scored 5. In that study, raw data showing how much the trauma symptoms changed was not provided (Osofsky et al., 2018). Overall, the relatively weaker areas were blinding, adherence, and accounting for confounders. Studies were not excluded based on quality, and all studies were retained based on their relevance to the research topic.

The results of data extraction are shown in Appendix 3, and a descriptive overview of the key findings of the included studies is provided below.

### What contexts and settings were these interventions implemented?

A summary of the settings of studies included in the review is displayed in Table 1, which includes the countries, types of natural disasters, populations, and the start times of interventions following the disasters. Three studies were conducted after the tsunami, six after the hurricane, one after flooding, and eight after an earthquake. Ages of the populations in these 18 studies were presented in various forms, including classifications by grades, age ranges, and average ages. Five studies reported grades of the participants, and they ranged from Grade 1 to Grade 8 (Cobham & McDermott, 2024; Dhital et al., 2019; Jaycox et al., 2010; Salloum & Overstreet, 2008; Wolmer et al., 2003); thirteen studies reported age ranges from 5 to 18 (Berger & Gelkopf, 2009; Cain et al., 2010; Catani et al., 2009; Fernandez, 2007; Giannopoulou et al., 2006; Goenjian et al., 2005; Graham et al., 2017; Osofsky et al., 2018; Plummer et al., 2008; Salloum & Overstreet, 2008; Trentini et al., 2018; Vijayakumar et al., 2006; Wolmer et al., 2005); and 11 studies provided participants’ average ages, with ranges from 8.2 to 15.7 (Cain et al., 2010; Cobham & McDermott, 2024; Dhital et al., 2019; Giannopoulou et al., 2006; Graham et al., 2017; Jaycox et al., 2010; Osofsky et al., 2018; Trentini et al., 2018; Wolmer et al., 2005; Wolmer et al., 2003). However, since Goenjian et al. (2021) was a 25-year follow-up study, the average ages of the participants were 36.18 for the intervention group and 38.24 for the control group at the time of follow-up.

**Intervention setting**. Of these 18 studies, eleven interventions were carried out in schools, which was the most common setting, three in communities, and others in refugee camps, clinics, and participants’ own homes. Some studies conducted their interventions in more than one location, according to their study designs.

**Start time of the interventions**. Eight studies targeted acute trauma exposure, which were conducted 0–6 months post-disaster. Three studies targeted 6–12 months post-disaster, and four targeted 1–2 years post-disaster. One study only mentioned that the intervention was conducted within 20 months of the disaster but did not specify when it started.

**Table 1:** Settings of the studies

Author (Year)	Region & Country	Disaster	Population (Age & *N*)		Context	Intervention Starting Time following the disaster	Length of the Intervention
Berger and Gelkopf (2009)	Welligama, Sri Lanka	Tsunami	9-14y	166	School	14 months	3 months
Cain et al. (2010)	Louisiana State, USA	Hurricane	5-15y, M = 9.5	99	School/FEMA trailer park	Within 20 months (did not specify)	6 weeks
Catani et al. (2009)	Vadamarachchi, Sri Lanka	Tsunami	8-14y	31	Refugee camp	3 weeks	2 weeks
Cobham and McDermott (2024)	Queensland, Australia	Floods	3–7 grades, M = 9.85	19	School	5 months	About 10–12 weeks
Dhital et al. (2019)	Dhading, Nepal	Earthquake	6–8 grades, M = 13	1220	School	17 months	N/A
Fernandez (2007)	Molise, Italy	Earthquake	7-11y	22	School	1 month	1 year
Giannopoulou et al. (2006)	Athens, Greece	Earthquake	8-12y, M = 9.6	17	Community	2 months (group 1) 4 months (group 2)	6 weeks
Goenjian et al. (2021)	Gumri, Armenia	Earthquake	M (treated) = 36.18, M (control) = 38.24	75	N/A	25-year long-term follow-up of Goenjian et al. (2005)	N/A
Goenjian et al. (2005)	Gumri, Armenia	Earthquake	15-17y	125	School	1.5 years	3 weeks
Graham et al. (2017)	Louisiana State, USA	Hurricane	8-17y, M = 12.16	112	School	4 months	Ranged from 2 months to 32 months depending on individual needs
Jaycox et al. (2010)	Louisiana State, USA	Hurricane	4–8 grades, M = 11.6	118	Clinics/ school	15 months	2 months for CBITS, 7 months for TF-CBT
Osofsky et al. (2018)	Louisiana State, USA	Hurricane	15-17y, M = 15.7	212	School/ community	9 months	1 year 10 months
Plummer et al. (2008)	Louisiana State, USA	Hurricane	6-15y	12	School	6 months	6 weeks
Salloum and Overstreet (2008)	Louisiana State, USA	Hurricane	2–6 grades, 7-12y	56	School/ home	4 months	10 weeks
Trentini et al. (2018)	Umbria, Italy	Earthquake	5-13y, M = 9.15	332	N/A	1 day (start of the outreach, triage and psychoeducation prior to EMDR)	3 weeks
Vijayakumar et al. (2006)	Chennai, India	Tsunami	11-14y	135	Community	11 months	6 months
Wolmer et al. (2005)	Adapazari, Turkey	Earthquake	9-17y, M = 11.5	287	N/A	3-year follow-up of Wolmer et al. (2003)	N/A
Wolmer et al. (2003)	Adapazari, Turkey	Earthquake	1–5 grades, M = 8.2	202	School	4 months	4 weeks

N/A = Not Available, M = Mean, N = Number of Population; *The exact number of population is not provided

**Fig. 2:**
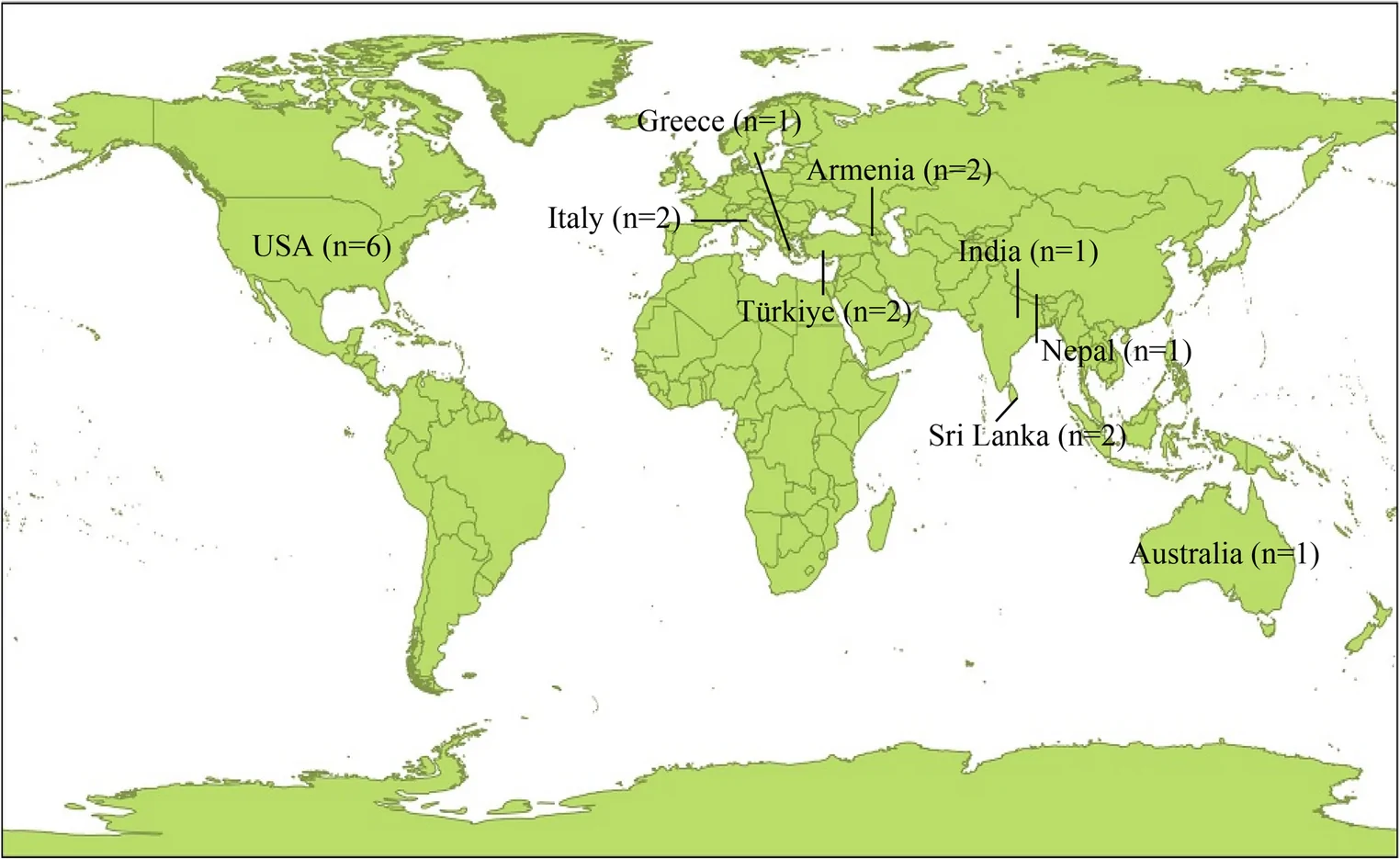
World map of where the studies were conducted

Figure 2 shows a map of where these studies were conducted. Two studies included were conducted in Sri Lanka, six in the United States, one in Australia, two in Armenia, two in Italy, one in Nepal, one in Greece, one in India and two in Türkiye. No study from South America or Africa was found.

### What Types of Child-Focused Psychosocial Interventions Targeting Stress Reactions Were Used During the First Two Years After a Natural Disaster?

A range of techniques and interventions were used to treat CYP who experienced PTSD or ASD following natural disasters, including Cognitive Behavioural Therapy (CBT; Cobham & McDermott, 2024; Giannopoulou et al., 2006; Goenjian et al., 2005; Graham et al., 2017; Jaycox et al., 2010; Salloum & Overstreet, 2008; Wolmer et al., 2003), Narrative Exposure Therapy (NET; Catani et al., 2009; Salloum & Overstreet, 2008), Psychological First Aid (PFA; Cain et al., 2010; Plummer et al., 2008), meditation-relaxation (MED-RELAX; Catani et al., 2009), play therapy (Vijayakumar et al., 2006), and Eye Movement Desensitisation and Reprocessing (EMDR; Fernandez, 2007; Trentini et al., 2018). Berger and Gelkopf (2009) implemented a programme called ERASE Stress, while Osofsky et al. (2018) conducted an intervention called the Youth Leadership Programme (YLP), but they did not mention what techniques the programmes involved. Also, Dhital et al. (2019) provided psychosocial support to CYP in a low-resource post-disaster environment. Details of interventions and specific implementation features can be found in Tables 2 and 3.

According to the stepped care levels proposed by Gibbs et al. (2021), psychological first aid (PFA) belongs to level 1, universal care (Cain et al., 2010; Plummer et al., 2008). Level 2 interventions include classroom-based cognitive behavioural interventions delivered by teachers (e.g., Berger & Gelkopf, 2009; Osofsky et al., 2018). Level 3 specialised interventions include EMDR delivered by professionals (e.g., Fernandez, 2007) and trauma-focused CBT (e.g., Graham et al., 2017). Although several interventions involve parents joining one or more of the sessions (e.g., Fernandez, 2007; Salloum & Overstreet, 2008), the interventions primarily adopted an individual symptom focus rather than a family system focus, except for Goenjian et al. (2005), where they specified that family interventions were provided when indicated.

**Table 2:** Specific implementation features and details of intervention with the TIDieR checklist (Items 1–6)

Author (Year)	Brief Name	Rationale	Materials	Procedures	Interventionist	Format
Berger and Gelkopf (2009**)**	ERASE Stress Program	The project avoided direct treatment of traumatic experiences, focusing instead on enhancing resilience and reinforcing resources. It incorporated cognitive performance skills, meditation, and vital energy exercises, and addressed traumatic experiences indirectly through narrative and art therapy.	The program provided psychological material and incorporated home assignments to try to engage the child’s caregivers.	The intervention included homework reviews, warm-up activities, experiential group activities, psychological educational lectures, practical coping skills training, and closing exercises, after which a new homework assignment was given.	Homeroom teachers	Group sessions conducted face to face. Each session included 12–16 students.
Cain et al. (2010)	PFA	Research on the effectiveness of PFA is relatively scarce, especially among children. This led to the initiation of this study.	N/A	The intervention provided psychoeducation on disaster reactions and coping strategies, guided emotional expression through verbal, written, and artistic activities, addressed intrusive or negative trauma-related thoughts, and taught skills for managing anxiety and displacement-related stress. Participants practiced relaxation and imagery techniques, engaged in activities to enhance self-esteem, understand anger, develop conflict-resolution skills, and reintroduce pleasurable routines.	licensed clinical social workers & graduate level social work student assistants	Face-to-face group sessions
Catani et al. (2009)	NET & MED-RELAX	NET is a brief trauma-focused intervention that meets the need of traumatized survivors and constructs a narrative that covers patients’ life and past traumatic experiences. MED-RELAX is proved to have promising results.	N/A	Children in NET group were asked to chronologically record their traumatic experiences and re-experience negative emotions. MED-RELAX began with psycho-education, followed by a brief assessment of children and concluded with breathing exercises for at least 15 min. The following contents included different relaxation and meditation skills and practices that were instructed by teachers in the same manner. Children were asked to practice meditation techniques for an hour a day as homework.	Teachers	Face-to-face.
Cobham and McDermott (2024**)**	TF-CBT	TF-CBT is one of the most evidence-based psychological interventions for youth exposed to any kind of traumatic events.	Both children and parents received workbooks to use in sessions.	Strategies taught included psycho-education about PTSS, cognitive restructuring, emotion regulation, behavioral activation and in vivo and imaginal exposure.	Clinicians	Individual and parents conjoint intervention.
Dhital et al. (2019)	Psychosocial support	In a low-resource post-disaster environment such as Nepal, teacher-led interventions may also be feasible and sustainable.	N/A	N/A	Teachers	Each school as a group by face-to-face.
Fernandez (2007**)**	EMDR	EMDR has been shown to be highly effective for PTSD and is the first choice of treatment for the population in schools.	N/A	The treatment was strictly carried out in accordance with the eight stages of EMDR: history taking, preparation and assessment, desensitisation, installation, body scan, closure and re-evaluation.	Members of mental health public agencies (ASL) and the EMDR Association	Face-to-face individual sessions. Parents were allowed to attend children’s sessions.
Giannopoulou et al. (2006)	CBT based on Teaching Recovery Techniques	Group therapy can serve a greater number of children in need, and group CBT in particular has been shown to be an effective psychosocial intervention for children with PTSD. Therefore, a clinic-based pilot study of group CBT intervention was designed and conducted.	N/A	The intervention consisted of seven stages: establishing group rules and providing psychoeducation on PTSD, teaching imagery techniques, identifying and regulating arousal symptoms, addressing avoidance behaviors, implementing exposure therapy (in Sessions 5 and 6), and concluding with summary, skill reinforcement, and relapse prevention.	Child and adolescent psychiatrists experienced in CBT & clinical psychologists trained in treating PTSD	Face-to-face group sessions.
Goenjian et al. (2021) [25-year follow-up of Goenjian et al. (2005)**]**	N/A	N/A	N/A	N/A	N/A	N/A
Goenjian et al. (2005)	TF-CBT & psychoeducation	N/A	N/A	Intervention included reconstructing earthquake related experience, linking trauma reminders with experience, identifying post-disaster stress and adversity, assisting with grief resolution by engaging in positive reminiscing and identifying missed developmental opportunities.	Psychiatric outreach program staff	Face-to-face individual and group interventions
Graham et al. (2017)	CBITS & TF-CBT	CBITS incorporates many CBT principles. TF-CBT is acknowledged as a well-supported intervention that has the greatest evidence of effectiveness in treating CYP post-disaster.	N/A	Interventions included cognitive processing, psychoeducation, trauma narratives, relaxation, social skills training, coping skills development and parent training.	Clinicians	Face-to-face individual session
Jaycox et al. (2010)	CBITS & TF-CBT	To examine the predictors of CYP performance in each type of intervention, in order to inform the future of post-disaster resource allocation.	N/A	Both interventions incorporated cognitive behavioral skills, including psychoeducation, trauma narratives, in vivo mastery of trauma reminders, relaxing, emotion regulation, cognitive coping skills and enhanced safety.	N/A	CBITS included individual and group interventions, TF-CBT included individual and parents conjoint intervention.
Osofsky et al. (2018)	The YLP	The YLP aimed to benefit the community by developing future leaders, enhancing students’ mental health and involving community members in youth activities.	N/A	Meetings and volunteering that helped improve students’ self-efficacy and reduce trauma-related symptoms.	Teachers & mental health professionals	Face-to-face group sessions
Plummer et al. (2008)	PFA	Research on the effectiveness of PFA is relatively limited.	N/A	N/A	Licensed clinical social workers	Face-to-face group sessions
Salloum and Overstreet (2008**)**	Project LAST (Drawing on principles from TF-CBT and narrative therapy for grief and trauma)	The theorized mechanisms of change included narrative exposure of trauma memories and development of positive coping strategies, as well as meaning-making.	N/A	The project included narrative exposure to trauma, development of in-depth and coherent narratives, positive coping strategies, as well as understanding and adaptation to losses.	Clinicians	Face-to-face individual and group interventions. Each group had 5 to 6 children.
Trentini et al. (2018)	EMDR - Integrative Group Treatment Protocol (EMDR-IGTP)	EMDR-IGTP draws on the core concepts of standard EMDR therapy, combining them in an adaptive form with a group and art therapy framework and incorporating interventions utilising the Butterfly Embrace, a self-help bilateral stimulation technique.	N/A	The intervention included 8 phrases: client history, preparation, assessment, desensitisation, installation, body scan, closure and reevaluation.	EMDR therapists	Group sessions online. Each group has 7–24 children.
Vijayakumar et al. (2006)	Play Therapy	Organised activities including playing, dancing, singing, painting, storytelling and drama are highly beneficial ways of expression for children and can be conducted in group settings.	Stones, pictures of illusion, puzzles, money and biscuits	Materials were used for addressing and acting out the emotions, learning anxiety management, discussing negative feelings, improving problem solving skills, saying good things about others to build relationships and experiencing peer pressure.	Three volunteers & two psychologists	Face-to-face group sessions.
Wolmer et al. (2005) [3-year follow-up of Wolmer et al. (2003)**]**	N/A	N/A	N/A	N/A	N/A	N/A
Wolmer et al. (2003)	School Reactivation Program based on a modified debriefing protocol	School- and teacher’s involvement is appropriate and scalable from an ecological perspective.	N/A	Intervention involved aspects of reconstruction of traumatic experiences, processing of intrusive thoughts, managing loss and death, and plans for the future.	Teachers	Each class as a group; face-to-face.

**Table 3:** Specific implementation features and details of intervention with the TIDieR checklist (Items 7–12)

Author (Year)	Setting	Duration	Tailoring	Modifications	Adherence monitoring (How Well [Planned))	Adherence (How Well [Actual)]
Berger and Gelkopf (2009**)**	School	The intervention groups participated in a total of 12 90-minute sessions (18 h in total).	N/A	N/A	During the first two sessions of the intervention, all teachers participated in two 3-hour supervision sessions facilitated by the trainer and assisted by two local professionals to ensure program fidelity, address issues that may arise, and monitor program adherence. As the trainer was unable to oversee the entire intervention process, further program fidelity was monitored through regular phone and web-based supervision.	Of those who completed the test, none dropped out. There was no missing data.
Catani et al. (2009)	Refugee camp	Intervention lasted for two weeks, including six sessions, each lasted for 60 to 90 min.	N/A	N/A	Each therapist offered the same number of treatments and filled out a detailed protocol after each session. Also, random observations of single session were carried out by psychologists and clinical supervisor.	No significant deviations from the treatment protocol were found. All the children finished the whole treatment module.
Cobham and McDermott (2024**)**	School	The parent program included two and the child program included eight weekly 90-minute sessions. The child program also comprised a booster session 2 to 4 weeks after the intervention.	N/A	N/A	All sessions were audio recorded to monitor treatment adherence for a randomly selected 20% of each session across clinicians and to facilitate the transcription of children’s trauma narratives.	Adherence was reported exemplary with all required content thoroughly covered and nothing additional or irrelevant included.
Dhital et al. (2019)	School	N/A	N/A	N/A	Clinical psychologists provided a two-day psychosocial support training for teachers, which consisted of a total of eight classes, each lasting one to two hours.	A total of 1,220 adolescents were recruited, of whom 1,070 completed the follow-up.
Fernandez (2007**)**	School	The treatment was divided into three cycles, one month, three months and one year after the event, with an average of 6.5 sessions per child.	Individual treatments for children range from 30 to 90 min, depending on each child’s developmental level and response.	N/A	At the end of each treatment cycle, clinicians would share and discuss.	Twenty-nine children received treatment, of whom three dropped out and four did not complete the full treatment program. The data analysis and conclusions are only for the 22 children who completed the full treatment program.
Giannopoulou et al. (2006)	The Community Mental Health Service	Total of six weeks of courses, two hours per week.	N/A	N/A	N/A	In total, 85% of the children completed the treatment, with 3 children in group 2 dropping out and not receiving the follow-up. They were not included in the final analysis.
Goenjian et al. (2021) [25-year follow-up of Goenjian et al. (2005)**]**	N/A	N/A	N/A	N/A	N/A	Seventy-five children participated in the follow-up study, 33 of whom participated in the intervention of Goenjian et al. (2005), and 42 children did not.
Goenjian et al. (2005)	School	Four 90-minute groups and, on average, two 1-hour individual treatments in 6 weeks.	Students with the most severe symptoms received four individual treatments.	N/A	Treatment was provided during school times.	There were 38 students participating in the treatment and 56 students who did not participate formed as the control group, but in the 5-year follow-up, there were only 36 students in the treatment group and 27 in the control group being analyzed.
Graham et al. (2017)	School	Weekly 55-minute sessions, each day 4 to 8 students received the treatment.	Clinicians tailored individualized treatment to the needs of each participant, and the timing and pace of treatment depended in part on CYP’s response, resulting in a different number of follow-up for each student (every three months until the end of the student’s treatment)	N/A	N/A	Some students stopped the treatment because of transfer in schools.
Jaycox et al. (2010)	Clinics/ School	CBITS contained 10 group sessions and 1 to 3 individual sessions. TF-CBT contained 12 individual or parents conjoint interventions.	N/A	N/A	N/A	In CBITS group, 98% of students started the treatment, 91% completed. In TF-CBT group, 23% of students started the treatment, 15% completed.
Osofsky et al. (2018)	School/ community	Meetings were held every week.	N/A	N/A	Facilitators met weekly with mental health professionals from the Louisiana State University Health Sciences Center Department of Psychiatry to discuss and ensure the focus of the project.	Only students from high school were assessed for their self-efficacy after the project.
Salloum and Overstreet (2008**)**	School/ home	The intervention lasted for ten weeks, each session lasted approximately one hour.	Children in group treatment had additional individual sessions to discuss trauma-related feelings and needs, while those in individual treatment extended their session time to cover similar topics.	N/A	All clinicians attended a weekly 3-hour supervision meeting to report on the implementation of the treatment program and to complete the adherence checklist.	There was 100% adherence for the group treatment and 97.35% adherence for the individual treatment. The dropout rate was 11%, and 7% of children did not attend the follow-up.
Trentini et al. (2018)	N/A	Children received EMDR-IGTP treatment once a week for 3 weeks and were tested before the start of the intervention and approximately 1 week after the end of the last intervention.	N/A	N/A	N/A	Some children did not complete a full treatment cycle, but they were excluded from the statistical analysis.
Vijayakumar et al. (2006)	Community	The treatment lasted for six months and consisted of six 2-hour sessions, each of which was repeated four times to make sure all children attended.	N/A	N/A	230 children attended the intervention and were reassessed 9 months after the intervention.	Only 150 children participated in the reassessment, 65 of whom finished the whole module and 15 children that participated in more than two sessions of the module. The remaining 70 children formed the control group.
Wolmer et al. (2005) [3-year follow-up of Wolmer et al. (2003)**]**	N/A	N/A	N/A	N/A	N/A	287 children participated in the follow-up study, 67 of whom participated in the intervention of Wolmer et al. (2003), and 220 children did not.
Wolmer et al. (2003)	School	The intervention lasted for four weeks, with two 2-hour meetings per week.	N/A	N/A	The teachers were in charge of the class activation and all the children participated.	N/A

**Psychological First Aid (PFA).** Plummer et al. (2008) is the pilot study of Cain et al. (2010), both of which held a 6-week session of PFA. The intervention provided psychoeducation, guided emotional expression, anxiety management, and intrusive thought management skills. Participants practised relaxation and imagery techniques, engaged in activities to enhance self-esteem, understand anger, and develop conflict-resolution skills. It is worth noting that the intervention was initially planned to be conducted as a pretest–posttest control group design but was changed to a pretest–posttest group design in the pilot study. Participants were treated in a group format.

**Trauma-focused CBT (TF-CBT).** The most commonly used intervention was TF-CBT, with 5 out of 15 studies adopting this approach. In terms of specific models used, Ehlers and Clark’s CT-PTSD model (Ehlers & Clark, 2000), TF-CBT from Cohen et al. (2012), the modified Teaching Recovery Technique (Smith et al., 1999), and CBITS were utilised (Ngo et al., 2008). Both individual and group CBT were delivered in the included studies. For individual CBT, TF-CBT was adopted and delivered by clinicians, including two parent sessions and eight child sessions, to treat children who met the criteria for PTSD or PTSS 4 months post-disaster (Cobham & McDermott, 2024). Key intervention components included psychoeducation about PTSS, cognitive restructuring, in-vivo and imaginal exposure, emotion regulation, and behavioural activation. Graham et al. (2017) also used TF-CBT and Cognitive Behavioural Intervention for Trauma in Schools (CBITS) to treat CYP individually. Participants with elevated PTSD symptoms, were randomly allocated to either group or individual forms of intervention for approximately one hour per session. Participants in group treatment had additional individual sessions to discuss trauma-related feelings and needs, while those in individual treatment extended their session time to cover similar topics. Jaycox et al. (2010) examined CBITS and TF-CBT among CYP with elevated PTSD symptoms in schools, where CBITS involved group and individual components, and TF-CBT involved individual and joint sessions with parents.

For group-based interventions, whilst Giannopoulou et al. (2006) described the intervention as a group intervention using CBT principles, the model they used was adopted from Teaching Recovery Techniques, which included exposure, thus incorporating trauma-focused elements. The intervention consisted of six weekly sessions, each lasting two hours. Participants were divided into two groups according to the timing of their treatment, namely 2 months and 4 months post-disaster. The intervention comprised seven stages: establishing group rules and providing psychoeducation on PTSD, teaching imagery techniques, identifying and regulating arousal symptoms, addressing avoidance behaviours, implementing exposure therapy (in Sessions 5 and 6), and concluding with a summary, skill reinforcement, and relapse prevention. Goenjian et al. (2005) conducted four 90-minute group sessions and, on average, two 1-hour individual treatments over a 6-week period with adolescents who met DSM-III criteria for PTSD 1.5 years after the earthquake, including psychoeducation and TF-CBT techniques.

**Narrative Exposure Therapy (NET).** Catani et al. (2009) conducted a RCT to compare the effectiveness of NET and MED-RELAX in a refugee camp in Sri Lanka. Both treatments consisted of six sessions, each lasting 60 to 90 min, completed within two weeks. CYP in the NET group were asked to chronologically record their traumatic experiences and re-experience negative emotions until their fear response was significantly reduced.

**Meditation-Relaxation**. In Catani et al. (2009)’s study, MED-RELAX began with psychoeducation, followed by a brief assessment of CYP, and concluded with breathing exercises lasting at least 15 min. The programme included various relaxation and meditation skills and practices taught by teachers in the same manner. CYP were instructed to practise meditation techniques for an hour daily as homework.

**Play Therapy**. The therapy examined by Vijayakumar et al. (2006) lasted six months, comprising six 2-hour sessions that addressed and enacted emotions using activities to learn anxiety management, discuss negative feelings by describing pictures, solve puzzles to improve problem-solving skills, say positive things about others to build relationships, and learn how to handle peer pressure against smoking and alcoholism.

**Eye Movement Desensitisation and Reprocessing (EMDR).** Both individual and group EMDR treatments were examined in the studies. Fernandez (2007) investigated the use of EMDR, dividing the treatment into three cycles: one month, three months, and one year after the disaster, with an average of 6.5 sessions per participant. The treatment was strictly carried out in accordance with the eight stages of EMDR. The duration of individual treatment varied according to each child’s developmental level and response, with parents permitted.

**Integrative Programmes.** Salloum and Overstreet (2008) examined Project LAST, an integrated intervention that drew upon principles of CBT, NET, and narrative therapy to develop a coherent narrative of trauma and loss. The research was conducted in three primary schools. Berger and Gelkopf (2009) examined ERASE Stress, a programme that included homework reviews, warm-up activities, experiential group activities, psychological educational lectures, practical coping skills training, and closing exercises. The intervention was delivered face-to-face in 12 weekly 90-minute sessions, each involving 12–16 students. Dhital et al. (2019) offered psychosocial support to students at different schools, and a total of 1,220 CYP were recruited, of whom 1,070 completed the follow-up. However, the study did not provide specific details regarding the intervention materials or implementation procedures. Osofsky et al. (2018) evaluated YLP, targeting high school students and facilitated by teachers and mental health professionals. The YLP encouraged students to participate in school and community service activities and aimed at reducing their trauma symptoms by enhancing their self-efficacy. Wolmer et al. (2003) and Wolmer et al. (2005) created a programme called the School Reactivation Programme that integrated a modified debriefing protocol combined with a psychoeducational module and cognitive-behavioural techniques, addressing topics on trauma, grief, and anger. These were facilitated by teachers through eight two-hour sessions.

### How Effective Were These Interventions in Achieving Their Intended Outcomes? Effective Were These Interventions in Achieving Their Intended Outcomes?

Studies adopted heterogeneous outcome measures, and not all studies reported effect sizes. Table 4 reports a narrative summary of the PTSD-related outcomes of interventions.

**Table 4:** Summary of the outcome of interventions

Author (Year)	Interventions	Outcome Measurements	Summary of findings
Berger and Gelkopf (2009**)**	ERASE Stress Program	UPID	PTSD scores in the ES-SL group showed significant improvement over time (*p* < .001). The probable PTSD diagnosis rate decreased dramatically in the ES-SL group (from 33.3% to 6.0%) compared to the waiting list group (WL) (31.7% to 28.1%); the difference in trajectory was significant (*p* = .001). Participants with probable PTSD at baseline experienced greater symptom reduction than those without (*p* = .006).
Cain et al. (20102010)	PFA	CPTSD-RI	Children showed a statistically significant reduction in PTSD symptoms (*p* = .027). This finding indicated that although PTSD symptoms improved significantly, the average symptom level remained within the moderate range.
Cobham and McDermott (2024**)**	TF-CBT	PTSD-RI	The mean total PTSD-RI symptom score decreased significantly from 47.95 (pre-treatment) to 7.00 (12-month follow-up). While 17 children met criteria for full or sub-clinical PTSD diagnosis pre-treatment, no children met criteria for either at the 12-month follow-up.
Dhital et al. (2019)	Psycho-social support	CPSS	The intervention did not improve mental health symptoms and hope among adolescents at 6 months follow-up. No significant difference in mean PTSD scores was observed between the intervention and control groups after the intervention (*p* = .477).
Fernandez (2007**)**	EMDR	A directly administered questionnaire prepared by the National Institute of Health which consists of a list of typical PTSD symptoms based on DSM-IV TR.	Overall PTSD symptoms were significantly reduced at the 0.01 level (*p* = .003), with statistically significant improvements in Avoidance (*p* = .015), Intrusiveness (*p* = .011), and Arousal (*p* = .015). Three months following the traumatic event (earthquake), the proportion of participants meeting PTSD criteria did not significantly decrease after the first treatment cycle; the proportion gradually declined over time, from an initial 61% to a final 9%.
Giannopoulou et al. (2006)	CBT	CRIES-13	ANOVA indicated significant changes in PTSD across different time points (*p* < .001). Pairwise comparisons showed a significant decrease in PTSD scores after treatment (*p* < .001), and at the 18-month follow-up (*p* < .001), but no significant change at the 4-year follow-up (*p* > .05).
Goenjian et al. (2021) [25-year follow-up of Goenjian et al. (2005)**]**	N/A	PTSD-RI & PCL	PTSD-RI scores of the intervention group were significantly lower than students in the control group (*p* < .05). The mean change of the treated group was significantly greater than that of the control group (*p* < .03). PCL scores of the treated group were less than the not-treated group (*p* < .02).
Goenjian et al. (2005)	TF-CBT & psycho-education	CPTSD-RI	A significant decrease in CPTSD-RI scores was observed in students 3.5 years after the treatment ended (*p* < .001). Scores of the students treated were significantly lower than those in the control group (*p* < .01).
Graham et al. (2017)	CBITS & TF-CBT	TSCC	TSCC showed a significant decrease in the total score (*p* < .001). Significant reduction was found in overall TSCC total scale from baseline to Follow-up 1 (*p* < .001) and to Follow-up 2 (*p* < .001), but not from Follow-up 1 to Follow-up 2.
Jaycox et al. (2010)	CBITS & TF-CBT	UPID & CPSS	Ten months after the treatment, the mean PTSD scores of the TF-CBT group shifted to the normal range, with those in the CBITS group falling into the lower clinical range. The proportion of children with PTSD symptom scores in the risk range (CPSS score ≥ 12) was 65% in the CBITS group and 43% in the TF-CBT group, which were comparable (*p* = .22).
Osofsky et al. (2018)	The YLP	Status Questionnaire for Students (SQS) & UPID	There were decreases in trauma symptoms in students in the YLP group, (*p* < .001); no change in trauma symptoms was observed in the non-YLP group.
Plummer et al. (2008)	PFA	The Reaction Index (a questionnaire based on CPTSD-RI)	There were no statistically significant differences in PTSD symptoms, *p* = .756.
Trentini et al. (2018)	EMDR-IGTP	CRIES-13	The overall effect of EMDR-IGTP was significant (Cohen’s *f²* = 1.19). Subgroup analysis showed that older children benefited more (*p* = .001) and girls showed greater symptom improvement than boys (*p* = .004). Earlier treatment also led to greater symptom improvement (*p* = .001).
Vijayakumar et al. (2006)	Play therapy	CPTSD-RI	Although PTSD symptoms decreased after treatment, it was not significant (*p* = .964) in the treatment and control group.
Wolmer et al. (2003)	Modified debriefing with CBT and psychoeducation content	TDGS & Turkish version of CPTSD-RI	The mean severity of posttraumatic stress (*p* < .001) and traumatic dissociation (*p* < .001) decreased significantly, while grief (*p* < .001) increased. The percentage of children with severe and very severe PTSS dropped from 30% to 18%, similar to the proportion of children not directly affected by the earthquake. 27.5% of the children experienced an increase in PTSS severity.
Wolmer et al. (2005)[3-year follow-up of Wolmer et al. (2003)**]**	N/A	CPTSD-RI & Traumatic Dissociation and Grief Scale (TDGS)	Significant decrease was observed in all three domains: posttraumatic stress (*p* < .02), dissociation (*p* < .001) and grief (*p* < .001). 29% of the children experienced an increase in PTSS severity, 30% remained at the same level, and 41% moved to a lower severity category. No significant differences in PTSD symptoms were found between the treatment and control group.

**TF-CBT.** Trauma-focused CBT interventions showed a significant reduction in PTSD symptoms measured by different questionnaires (e.g. PTSD-RI, CRIES-13) across multiple studies compared to control (e.g. Cobham & McDermott, 2024; Giannopoulou et al., 2006; Goenjian et al., 2005). One study demonstrated a significant difference between adolescents in the treatment versus control group in terms of the mean decrease in PTSD symptoms at 5 years and 25 years follow-up (Goenjian et al., 2005, 2021).

**Eye Movement Desensitisation and Reprocessing**. Fernandez (2007) found that overall PTSD symptoms were significantly reduced (*p* = .003). Similarly, results from Trentini et al. (2018) demonstrated a significant overall effect of EMDR-IGTP on CRIES-13 scores (Cohen’s f² = 1.19). Greater symptom reductions were observed in participants who received earlier treatment (*p* = .001, η²*p* = .12), in older children (*p* = .001, η²*p* = .36), and in girls compared to boys (*p* = .004, η²*p* = .04).

**Integrated Programmes**. Berger and Gelkopf (2009) found that PTSD scores in the ES-SL group showed significant improvement over time (*p* < .001). The probable PTSD diagnosis rate decreased dramatically from 33.3% to 6.0% in the ES-SL group, whereas the WL group showed minimal change (31.7% to 28.1%), with three people reaching the clinical threshold of PTSD. A chi-square test revealed a significant difference in the trajectory of PTSD changes between groups (χ² = 14.02, *p* = .001). In addition, a strong positive correlation was found between baseline PTSD scores and the degree of improvement (*r* = .70), and those with probable PTSD experienced greater symptom reduction than those without (*p* = .006). Osofsky et al. (2018) reported decreases in trauma symptoms in students in the YLP, and this change was significantly associated with the increase of their self-efficacy (*p* < .001). However, raw data on how much the trauma symptoms changed was not provided. Dhital et al. (2019) revealed a non-significant difference between the intervention group (delivered by teachers) and control group on PTSD symptoms, with authors postulating that teacher-mediated interventions alone might not be sufficient to address the students’ mental health concerns.

Of note, Wolmer et al. (2003, 2005) revealed mixed findings regarding the effectiveness of their intervention. In the short term, the intervention appeared to be partially effective: the proportion of CYP with severe or very severe PTSS decreased from 30% to 18% (*p* < .001). However, grief symptoms increased during this period (*p* < .001), and 27.5% of children experienced a worsening of PTSS symptoms. Over a three-year follow-up, there were further overall improvements—post-traumatic stress (*p* < .02), dissociation (*p* < .001), and grief (*p* < .001)—all showed significant reductions compared to immediately after treatment. Nonetheless, 18% of participants continued to report severe PTSD symptoms, and crucially, there was no significant difference in outcomes between the treatment and control groups. This suggests that while some symptom improvement was observed over time, the long-term effectiveness of the intervention may have been limited or indistinguishable from natural recovery processes.

**Studies with an active comparative group.** Jaycox et al. (2010) reported that, 10 months after the treatment, the proportion of children with PTSD symptom scores in the risk range (Child PTSD Symptom Scale score ≥ 12) was 65% in the CBITS group and 43% in the TF-CBT group, which were comparable. Catani et al. (2009) found that both NET and MED-RELAX treatments had a main effect of time on PTSD symptoms. No significant difference between the effects of the two treatment modalities was observed across time points. In another study, no significant difference was found between group and individual therapy, where a significant main effect in reduction of PTSD symptoms was observed from pre-treatment to post-treatment (*p* = .001, d = 1.16) and at follow-up (*p* = .001, d = 1.82) (Salloum & Overstreet, 2008). Also, the age × gender interaction for PTSD symptoms approached significance (*p* = .054, partial η² = 0.082), with younger girls (7–9 years) showing the least improvement compared to younger boys (7–9 years), older girls (10–12 years), and older boys (10–12 years).

**Play Therapy**. For play therapy, Vijayakumar et al. (2006) conducted a study on play therapy delivered by trained volunteers and found a non-significant result between the treatment and control groups (*p* = .964). Although caution is needed, as the trial was a non-randomised study.

## Discussion

The review aimed to evaluate the contexts and settings of psychological interventions implemented for addressing stress reactions in CYP within two years after the occurrence of natural disasters; the types of interventions targeting stress reactions, and their effectiveness in achieving their intended outcomes. Overall, studies have shown that treating PTSD after natural disasters for CYP in different settings has been largely effective in reducing PTSD symptoms. It is worth noting that none of the studies covered children younger than five years old, which means that based on the current review, it is difficult to recommend evidence-based approaches for younger children.

### Context

It is worth noting that some interventions were delivered by non-professionals such as school teachers and community volunteers, rather than qualified mental health professionals. This is called “task-sharing” and “task-shifting”, which is especially important in low-resource settings where there are few trained clinicians. Eight out of the 16 studies were conducted in Asian countries. This may be because Asia has the highest frequency of natural disasters (Tin et al., 2024), being prone to earthquakes, tsunamis, floods, and having a high population density that increases the number of people affected by natural disasters as well as the mortality rate (Shaw & Team, 2009). Twelve out of 16 studies were conducted at schools. From an ecological perspective, aside from the home, school is the most natural support system for CYP, providing therapists with the opportunity to observe and follow up on their mental states. Additionally, teachers and schoolmates are regarded as important predictors of reducing severe PTSD symptoms (Wolmer et al., 2003). However, Le Roux et al. (2022) also pointed out that interventions at schools may exclude CYP who are unable to attend school. Therefore, it is essential for agencies and centres that provide psychological interventions to consider those who face unequal educational opportunities and support them to access the treatment they need. Interventions provided in the community can bridge the gap to compensate for the lack of school-based interventions.

### Intervention Type

The interventions adopted include both trauma-focused treatments and non-trauma-focused treatments, manualised interventions such as CBT, NET, PFA, and EMDR, as well as bespoke, integrative interventions. The ERASE programme avoided direct treatment of traumatic experiences, focusing instead on enhancing resilience and reinforcing resources. The programme incorporated cognitive performance skills, meditation, and vital energy exercises, and addressed traumatic experiences indirectly through narrative and art therapy. The YLP was a programme developed specifically to restore the health of adolescents after Hurricane Katrina. Therefore, it may not be generalisable in other situations. However, the participatory action principles included in the YLP allowed for tailoring the programme to different populations or disasters in different contexts to achieve improvements (Osofsky et al., 2018). Project LAST, which included TF-CBT as well as NET and specifically targeted CYP from elementary schools who had experienced grief and trauma caused by disaster, violence, and death, was applied by Salloum and Overstreet (2008) to students in grades two to six without explaining the reasons for the exclusion of the first year. All other treatments were widely used in CYP ranging from preschool to 18-year-old CYP. In addition, Catani et al. (2009), Cobham and McDermott (2024), Fernandez (2007), Plummer et al. (2008), and Giannopoulou et al. (2006) only included fewer than 40 children, and such small sample sizes may limit the generalisability of the results.

In terms of the forms of treatments, Salloum and Overstreet (2008) did not find any differential dropout rate between group and individual intervention. It is worth noting that although Le Roux and Cobham (2022) stated in their review that there was a lack of clear and high-quality evidence comparing the effectiveness of group therapy with that of individual therapy, the present review found that many studies adopted a group format.

### Timing and Effectiveness

The present scoping review focuses on interventions commenced within two years after the disaster, due to the criticality and sensitivity of the early post-traumatic period. We observed that the two interventions which did not yield significant reductions in PTSD symptoms took place one year after the disaster. It is possible that the timing of implementation could influence the effectiveness of the intervention. Nevertheless, the type of intervention might also influence the outcomes, as one intervention was psychosocial support (Dhital et al., 2019) and the other was play therapy (Vijayakum et al., 2006), which could differ in terms of the hypothesised mechanisms of change and dosage when compared to trauma-focused interventions. No studies directly examined the effect of timing on the intervention’s effectiveness. Therefore, we could not determine whether there was an optimal intervention timing post-disaster. The current findings align with the existing literature (Newman et al., 2014), suggesting that timing as a moderator of effectiveness remains a debated and unresolved topic. Feasibility should also consider factors such as the availability of providers, safety, and funding, as these can influence the timing of the intervention. This highlights the importance of disaster preparedness and the integration of MHPSS into early disaster response.

Overall, greater reductions in PTSD symptoms were observed than in natural recovery, which is consistent with that of Newman et al. (2014). However, some participants were found to have increased symptoms or to have been revictimised after the treatment, which was explained as possibly being due to those CYP having previously been exposed to other traumas or stressors (Jaycox et al., 2010; Wolmer et al., 2003). Due to the heterogeneity of sample populations (number, age, presence of other co-morbid illnesses, level of trauma exposure, and socio-cultural background), as well as differences in the design of interventions (treatment initiation and duration, diagnostic and measurement tools, and qualification of therapists) (Cobham & McDermott, 2024), it is not possible to make a direct comparison between different studies or determine which type of intervention is most effective.

Currently, there is no clear evidence comparing different interventions, except for the study by Catani et al. (2009), which compared NET and MED-RELAX. The study found both interventions had similar effects across time points during the treatment, but MED-RELAX may have a stronger long-term effect on treating CYP’s PTSD compared to NET. This study had high adherence and was of high quality, but its results were in contrast with research on adult populations, which indicated that NET had more obvious advantages in the immediate aftermath of trauma (Bisson et al., 2007). Integrative interventions may be beneficial, such as NET and CBT (Salloum & Overstreet, 2008), but it is not yet possible to draw a definitive conclusion regarding which component plays a major role. Psychoeducation is often offered in conjunction with other forms of intervention, particularly CBT. However, there are still conflicting findings on the effectiveness of psychoeducation. While some studies have shown that psychoeducation as an adjunctive therapy can lead to better outcomes, there are also studies that have not documented the benefits associated with it (Akena et al., 2021). The present review did not find any studies using psychoeducation as a stand-alone intervention, which makes it difficult to determine the specific effects of psychoeducation. Further research needs to examine psychoeducation separately to clarify its effectiveness and possible scope of application.

In terms of negative findings, Dhital et al. (2019) and Vijayakumar et al. (2006) did not find significant improvements in reducing PTSD symptoms. Vijayakumar et al. (2006) suggested that this could be because CYP may have been more accepting of their PTSD after receiving the intervention, resulting in a higher retest score that created the illusion of no improvement. Vijayakumar et al. (2006) argued that targeted, indicated interventions might be more effective. At the same time, Dhital et al. (2019) reported that the psychosocial support provided by teachers showed no significant effect. They argued that the result could possibly be due to the interventionists being teachers who were not mental health professionals. Although significant differences between groups were found by Wolmer et al. (2003), Wolmer et al. (2005) revealed that there was no group difference. They suggested that the long-term clinical course of post-disaster symptoms might be affected by other characteristics such as pre-disaster vulnerability, family life, and support systems rather than participation in the intervention programme.

It is important to consider the applicability of interventions across different cultures. For example, the reason why Catani et al. (2009) successfully conducted MED-RELAX in Sri Lanka and demonstrated its effectiveness may be related to the cultural significance of meditation in Tamil culture, which facilitated CYP’s adaptation to this treatment. In other cultures, however, MED-RELAX may not be equally effective. Conversely, the limited success of play therapy in India may be attributed to cultural, economic, or perceptual differences, which could lead to outcomes that differ from those observed in other countries.

As the primary recommended intervention by both the APA (2020) and NICE guidelines (2018), CBT, specifically TF-CBT, has been shown to be the most well-documented and frequently used psychological intervention in the treatment of youth exposed to any type of traumatic event (Cobham & McDermott, 2024; Graham et al., 2017), and the results of all studies included in the present review support this assertion. In particular, the effects of CBT were reported to last up to 25 years (Goenjian et al., 2005), but because there was only one study with such a long follow-up, the reliability of this conclusion has not been fully established. Conducting similar long-term follow-up studies in diverse disaster settings or populations in the future would help to strengthen the evidence. It has been reported that both TF-CBT and CBITS can reduce PTSD. According to Jaycox et al. (2010), TF-CBT involves joint sessions with both parent and child, whereas CBITS is delivered in group settings for children only. Also, TF-CBT allows for individualised support, making it particularly effective for addressing avoidance symptoms and constructing trauma narratives. By contrast, CBITS may be a more practical and socially acceptable option, as it helps to reduce stigma and logistical challenges (Jaycox et al., 2010). That said, given the heterogeneity of the studies, caution is needed when inferring the superiority of the interventions.

PFA is regarded as the acute intervention of choice when treating individuals affected by disasters (Plummer et al., 2008). However, relatively few studies evaluating PFA met the inclusion criteria of the present review, and the two included studies yielded mixed findings, suggesting that its effects on PTSD symptom reduction may be modest. Importantly, PFA is a non-trauma-focused treatment that has been designed primarily to promote stabilisation, safety, and offer support, rather than to directly target trauma-related symptoms (World Health Organization, War Trauma Foundation, & World Vision, 2011). As such, PFA may function more effectively as an initial or supportive intervention rather than a standalone treatment for PTSD, particularly among children. In addition, due to the persistence of disasters, many children were unable to access immediate and professional assistance services; PFA may be helpful for acute recovery (Cain et al., 2010).

It is worth noting that we did not find any RCTs for another commonly used intervention for trauma, EMDR, recommended as a second-line treatment for PTSD by the NICE guidelines (2018). Only two relevant studies—Fernández (2007) and Trentini et al. (2018)—were found, both of which explored EMDR as an early intervention for treating CYP with PTSD within two years following a natural disaster.

Other than psychological interventions, psychosocial interventions such as play therapy in the community have also been used to support CYP (Vijayakumar et al., 2006), as playing games is considered one of the most effective methods for them to cope with stress. Under these circumstances, playworkers often take on the role of informal therapists, who are regarded as crucial in facilitating the positive adaptation of CYP with mild to moderate difficulties following exposure to mass trauma (Maria & Ian, 2022). As trusted adults, their roles are not only to create a positive atmosphere for CYP but also to actively support and help them address trauma, increase their mental resilience, and promote their overall health and development. However, it must be mentioned that only one of the included studies concerns play therapy (Vijayakumar et al., 2006), and it was noted that the therapy did not demonstrate a significant reduction in PTSD symptoms. This may be due to the intervention’s lack of specificity, as well as insufficient testing and validation of its effectiveness (Vijayakumar et al., 2006).

Additionally, it is important to note that psychosocial interventions have a more complex rehabilitation process than psychological interventions. This is because they not only involve the individual’s psychological state but also encompass elements of the entire social environment, and therefore require more support, resources, and time. These factors are interdependent and constraining (Kunz, 2009), making psychosocial interventions more challenging and difficult to implement.

Last but not least, interventions developed for specific disasters or populations, such as the ERASE Stress programme (Berger & Gelkopf, 2009) and the YLP (Osofsky et al., 2018), have alleviated trauma symptoms from novel perspectives and demonstrated associations with skill development and self-efficacy. Although these targeted interventions may lack a solid theoretical foundation or robust empirical evidence and may not be generalisable to other contexts or disasters, they can nonetheless offer valuable insights and foundations for future research and practice. Therefore, they merit greater attention and further investigation.

### Developmental Considerations

Despite differences in manifestations of PTSD symptoms across the developmental trajectory (Scheeringa et al., 2011), most studies did not consider or observe any interaction between age and the results. Except for Salloum and Overstreet (2008) and Trentini et al. (2018), where there was a significant or near-significant interaction between the effect and age. These two studies both suggested that the improvement would be greater with older age. This might be because, as children grow older, their cognitive maturity, abstract thinking ability, and language comprehension skills develop, making it easier for them to understand the intervention and transfer the learning to daily situations (Steinberg, 2014). This shows that different interventions may be needed across ages and that whole-school approaches/school-based interventions may need to be tailored and will not be a one-size-fits-all. A critical limitation in current literature is the lack of research on children under five years old. None of the studies included in this review covered this age group, making it difficult to recommend evidence-based approaches for very young children. Future research must prioritise age-specific effects and tailor interventions to the distinct cognitive and behavioural attributes of each developmental stage to ensure that PTSD treatments are both developmentally appropriate and effective.

### Clinical and Research Implications

CBT is the most used intervention technique and seems to be an appropriate treatment to help CYP following a natural disaster, depending on their actual condition. At the same time, given that CYP from different cultural backgrounds respond to trauma and accept treatment differently, and that economic conditions vary across countries or regions, it is important to develop specific interventions that are locally adapted, culturally sensitive, and cost-effective (Vijayakumar et al., 2006).

Moreover, a direct comparison of different interventions is currently still lacking in this field, and it is recommended that future studies design more comparative trials to directly compare the effects of different interventions to provide clearer guidance. This may also be more ethical than no-treatment control studies in emergency contexts, where both groups receive support. Although the interventions were based on different modalities (e.g., psychological first aid, debriefing, CBT), they appear to include similar components. This may also lead to confusion regarding which interventions are superior to others, as common elements may be involved, especially in integrated interventions. Future studies should clearly document the intervention content to allow reviews to distill the active ingredients.

Interventions in the early stages after a disaster are critical to the development of CYP, but the optimal timing and duration for conducting interventions have not been adequately studied. Therefore, future studies could focus on and explore the effects of interventions conducted at different time points after a disaster to determine the most effective time frame for interventions to maximise their effectiveness. Furthermore, based on the stepped care model, most studies focused on individual symptoms rather than a family approach. Future research could explore the effectiveness of family interventions.

Earthquakes have been the most studied common natural disaster. Surprisingly, floods, as the most frequent natural disasters in the world (World Health Organization, n.d.), have only been studied by Cobham and McDermott (2024) for early intervention in CYP after disaster. Thus, future studies are recommended to examine interventions for CYP exposed to floods.

An important point to note is negative outcomes and adverse events. For example, Wolmer et al. (2003) observed that grief increased after the intervention, and at follow-up, 27.5% of the CYP experienced an increase in post-traumatic stress severity. Whilst grief might not be a direct focus of the intervention, clinicians need to attend to increases in symptom severity and adjust the intervention accordingly. Future research should systematically document such events.

### Strengths and Limitations

This review also has some limitations. Whilst it is important to understand the effects of interventions in the early phases after a disaster, the same intervention might work differently when implemented at different times, and the needs immediately after the disaster might be different from those one year later. At this stage, we were unable to unpick such differences. Also, due to the nature of scoping reviews, low-quality studies were not excluded, even though a critical appraisal was done at the time of screening. This review covers a wide range of studies, up to 20 years, and it must be acknowledged that some of the interventions may have been updated. Due to the heterogeneity of the outcome measures and interventions used, a qualitative synthesis rather than a meta-analysis was conducted. Whilst children experience a wide array of psychological responses following a natural disaster, the current review only focuses on trauma and stress-related responses and trauma-related outcomes. It is important to note that some interventions might offer transdiagnostic benefits, although this is beyond the scope of the current review. The current review is also unable to tease apart the influence of hazard type on intervention effectiveness and feasibility, as some hazards may be more widespread and can influence the implementation of the interventions. Nevertheless, to our knowledge, this systematic scoping review is the first to focus on interventions for PTSD in CYP specifically within two years following natural disasters, which summarises the available relevant evidence and its shortcomings. This can help advance the field by understanding the phases in which the interventions are most helpful, supporting the development of a strategic emergency response from acute crisis to post-crisis recovery.

In conclusion, although psychological interventions for CYP’s PTSD within two years after a natural disaster have received some attention, they are still under-researched. Thus, more high-quality and updated relevant research is needed in order to better understand and improve the effectiveness of these interventions to help address the mental health status of CYP.

## Supplementary Information

Below is the link to the electronic supplementary material.

Supplementary Material 1 (DOCX 60.4 KB)

## Data Availability

No datasets were generated or analysed during the current study.
