# Supplementary material for: Psychological Interventions for PTSD Children and Adolescents Within Two Years of Natural Disasters: A Systematic Scoping Review
Source: J Child Adolesc Trauma. 2026 Mar 17;19(3):1559–80. doi: 10.1007/s40653-026-00847-w (PMC13546279; doi:10.1007/s40653-026-00847-w)
Supplement: Supplementary file 1 [file 40653_2026_847_MOESM1_ESM.docx]

# Appendices

## Appendix 1. Databases Search Strategies

| **Databases** | **Search Terms** |
| --- | --- |
| Embase | (treatment* **OR** intervention* **OR** therap* **OR** psychosocial intervention* **OR** psychological intervention* **OR** psychological first aid **OR** mental health first aid **OR** (Eye Movement Desensitisation and Reprocessing) **OR** critical incident stress management* **OR** debriefing).mp. [mp=title, abstract, heading word, drug trade name, original title, device manufacturer, drug manufacturer, device trade name, keyword heading word, floating subheading word, candidate term word] **OR** exp treatment outcome/ **OR** exp psychosocial intervention/ **OR** exp psychosocial care/ **OR** exp psychotherapy/ **OR** exp crisis intervention/ **OR** exp psychological first aid/ **OR** exp "eye movement desensitization and reprocessing"/ **AND** (immediate exposure* **OR** acute exposure* **OR** post-disaster).mp. [mp=title, abstract, heading word, drug trade name, original title, device manufacturer, drug manufacturer, device trade name, keyword heading word, floating subheading word, candidate term word] **AND** (natural disaster* **OR** earthquake* **OR** tsunami **OR** tornado **OR** flooding **OR** cyclone* **OR** wildfire* **OR** landslide* **OR** volcanic eruption* **OR** storm*).mp. [mp=title, abstract, heading word, drug trade name, original title, device manufacturer, drug manufacturer, device trade name, keyword heading word, floating subheading word, candidate term word] **OR** exp natural disaster/ **OR** exp hurricane/ **OR** exp earthquake/ **OR** exp tsunami/ **OR** exp tornado/ **OR** exp flooding/ **OR** exp wildfire/ **OR** exp landslide/ **OR** exp volcano/ **OR** exp "storm (weather)"/ **AND** (trauma* **OR** mental health **OR** stress **OR** Post-Traumatic Stress Disorder*).mp. [mp=title, abstract, heading word, drug trade name, original title, device manufacturer, drug manufacturer, device trade name, keyword heading word, floating subheading word, candidate term word] **OR** exp mental health/ **OR** exp posttraumatic stress disorder/ **AND** (family* **OR** child* **OR** adolesc* **OR** teen* **OR** young people).mp. [mp=title, abstract, heading word, drug trade name, original title, device manufacturer, drug manufacturer, device trade name, keyword heading word, floating subheading word, candidate term word] **OR** exp family/ **OR** exp family life/ **OR** exp child/ **OR** exp adolescent/ |
| MEDLINE | (treatment* **OR** intervention* **OR** therap* **OR** psychosocial intervention* **OR** psychological intervention* **OR** psychological first aid **OR** mental health first aid **OR** (Eye Movement Desensitisation and Reprocessing) **OR** critical incident stress management* **OR** debriefing).mp. [mp=title, book title, abstract, original title, name of substance word, subject heading word, floating sub-heading word, keyword heading word, organism supplementary concept word, protocol supplementary concept word, rare disease supplementary concept word, unique identifier, synonyms, population supplementary concept word, anatomy supplementary concept word] **OR** exp Treatment Outcome/ **OR** exp Psychotherapy/ **OR** exp Crisis Intervention/ **OR** exp Psychosocial Intervention/ **OR** exp Cognitive Behavioral Therapy/ **OR** exp Psychological First Aid/ **OR** exp Eye Movement Desensitization Reprocessing/ **AND** (immediate exposure* **OR** acute exposure* **OR** post-disaster).mp. [mp=title, book title, abstract, original title, name of substance word, subject heading word, floating sub-heading word, keyword heading word, organism supplementary concept word, protocol supplementary concept word, rare disease supplementary concept word, unique identifier, synonyms, population supplementary concept word, anatomy supplementary concept word] **AND** (natural disaster* **OR** earthquake* **OR** tsunami **OR** tornado **OR** flooding **OR** cyclone* **OR** wildfire* **OR** landslide* **OR** volcanic eruption* **OR** storm*).mp. [mp=title, book title, abstract, original title, name of substance word, subject heading word, floating sub-heading word, keyword heading word, organism supplementary concept word, protocol supplementary concept word, rare disease supplementary concept word, unique identifier, synonyms, population supplementary concept word, anatomy supplementary concept word] **OR** exp Cyclonic storms/ **OR** exp Earthquakes/ **OR** exp COVID-19/ **OR** exp Natural Disasters/ **OR** exp Tsunamis/ **OR** exp Tornadoes/ **OR** exp Floods/ **OR** exp Wildfires/ **OR** exp Landslides/ **OR** exp Volcanic Eruptions/ **AND** (trauma* **OR** mental health **OR** stress **OR** Post-Traumatic Stress Disorder*).mp. [mp=title, book title, abstract, original title, name of substance word, subject heading word, floating sub-heading word, keyword heading word, organism supplementary concept word, protocol supplementary concept word, rare disease supplementary concept word, unique identifier, synonyms, population supplementary concept word, anatomy supplementary concept word] **OR** exp Mental Health/ **OR** exp Stress Disorders, Post-Traumatic/ **OR** exp Stress Disorders, Traumatic, Acute/ **OR** exp Stress, Psychological/ **AND** (family* **OR** child* **OR** adolesc* **OR** teen* **OR** young people).mp. [mp=title, book title, abstract, original title, name of substance word, subject heading word, floating sub-heading word, keyword heading word, organism supplementary concept word, protocol supplementary concept word, rare disease supplementary concept word, unique identifier, synonyms, population supplementary concept word, anatomy supplementary concept word] **OR** exp Family/ **OR** exp Child, Preschool/ **OR** exp Child/ **OR** exp Adolescent/ |

| Databases | Search Terms |
| --- | --- |
| PsycInfo | treatment* **OR** intervention* **OR** therap* **OR** psychosocial intervention* **OR** psychological intervention* **OR** psychological first aid **OR** mental health first aid **OR** (Eye Movement Desensitisation and Reprocessing) **OR** critical incident stress management* **OR** debriefing).mp. [mp=title, abstract, heading word, table of contents, key concepts, original title, tests & measures, mesh word] **OR** exp Trauma Treatment/ **OR** exp Treatment/ **OR** exp Crisis Intervention Services/ **OR** exp Crisis Intervention/ **OR** exp Treatment Outcomes/ **OR** exp Psychotherapy/ **OR** exp Treatment Effectiveness Evaluation/ **OR** exp Cognitive Behavior Therapy/ **OR** exp Intervention/ **OR** exp Psychosocial Interventions/ **OR** exp Psychological First Aid/ **OR** exp Eye Movement Desensitization Therapy/ **OR** exp Stress Management/ **OR** exp "Debriefing (Psychological)"/ **AND** (immediate exposure* **OR** acute exposure* **OR** post-disaster).mp. [mp=title, abstract, heading word, table of contents, key concepts, original title, tests & measures, mesh word] **AND** (natural disaster* **OR** earthquake* **OR** tsunami **OR** tornado **OR** flooding **OR** cyclone* **OR** wildfire* **OR** landslide* **OR** volcanic eruption* **OR** storm*).mp. [mp=title, abstract, heading word, table of contents, key concepts, original title, tests & measures, mesh word] **OR** exp Natural Disasters/ **AND** (trauma* **OR** mental health **OR** stress **OR** Post-Traumatic Stress Disorder*).mp. [mp=title, abstract, heading word, table of contents, key concepts, original title, tests & measures, mesh word] **OR** exp Trauma/ **OR** exp Mental Health/ **OR** exp Acute Stress/ **OR** exp Posttraumatic Stress Disorder/ **OR** exp Stress/ **OR** exp Acute Stress Disorder/ **OR** exp Posttraumatic Stress/ **AND** (family* **OR** child* **OR** adolesc* **OR** teen* **OR** young people).mp. [mp=title, abstract, heading word, table of contents, key concepts, original title, tests & measures, mesh word] **OR** exp Family/ **OR** exp Adolescent Development/ **OR** exp Childhood Development/ |
| PubMed | (treatment*[Title/Abstract] **OR** intervention*[Title/Abstract] **OR** therap*[Title/Abstract] **OR** psychosocial intervention*[Title/Abstract] **OR** psychological intervention* [Title/Abstract] **OR** psychological first aid[Title/Abstract] **OR** mental health first aid[Title/Abstract] **OR** Eye Movement Desensitisation and Reprocessing[Title/Abstract] **OR** EMDR[Title/Abstract] **OR** critical incident stress management*[Title/Abstract] **OR** debriefing[Title/Abstract]) **AND** ((immediate exposure*[All Fields] **OR** acute exposure* [All Fields] **OR** post-disaster[All Fields]) **AND** (natural disaster*[Title/Abstract] **OR** earthquake*[Title/Abstract] **OR** tsunami[Title/Abstract] **OR** tornado[Title/Abstract] **OR** flooding[Title/Abstract] **OR** cyclone*[Title/Abstract] **OR** wildfire* [Title/Abstract] **OR** landslide*[Title/Abstract] **OR** volcanic eruption*[Title/Abstract] **OR** storm*[Title/Abstract]) **AND** (family* [Title/Abstract] **OR** child*[Title/Abstract] **OR** adolesc* [Title/Abstract] **OR** teen*[Title/Abstract] **OR** young people[Title/Abstract]) **AND** (trauma*[Title/Abstract] **OR** mental health[Title/Abstract] **OR** stress[Title/Abstract] **OR** postTraumatic Stress Disorder*[Title/Abstract]) |
| Scopus | TITLE-ABS-KEY("treatment*" **OR** "intervention*" **OR** "psychosocial intervention*" **OR** "psychological intervention*" **OR** "psychological first aid" **OR** "mental health first aid" **OR** "Eye Movement Desensitisation and Reprocessing" **OR** "EMDR" **OR** "critical incident stress management*" **OR** "debriefing" **OR** "therap*") **AND** ALL ("immediate exposure*" **OR** "acute exposure*" **OR** "post-disaster") **AND** TITLE-ABS-KEY ("natural disaster*" **OR** "earthquake*" **OR** "tsunami" **OR** "tornado" **OR** "flooding" **OR** "cyclone*" **OR** "wildfire*" **OR** "landslide*" **OR** "volcanic eruption*" **OR** "storm*") **AND** TITLE-ABS-KEY ("child*" OR "adolesc*" **OR** "teen*" **OR** "young people") **AND** TITLE-ABS-KEY ("trauma*" **OR** "mental health" **OR** "stress" **OR** "Post-Traumatic Stress Disorder*") **AND** ( LIMIT-TO ( DOCTYPE , "ar" ) ) **AND** ( LIMIT-TO ( LANGUAGE , "English" )) |

## Appendix 2. Mixed Methods Appraisal Tool, Version 2018 (Hong et al., 2018)

| Category of study designs | Methodological quality criteria | Responses | | | | | | | | | | | | | | | | | |  |
| --- | --- | --- | --- | --- | --- | --- | --- | --- | --- | --- | --- | --- | --- | --- | --- | --- | --- | --- | --- | --- |
|  |  | Berger & Gelkopf (2009) | Cain et al. (2010) | Catani et al. (2009) | Cobham & McDermott (2024) | Dhital et al. (2019) | Fernandez (2007) | Giannopoulou (2006) | Goenjian et al. (2021) | Goenjian et al. (2005) | Graham et al. (2017) | Jaycox et al. (2010) | Osofsky et al. (2018) | Plummer et al. (2008) | Salloum & Overstreet (2008) | Trentini et al. (2018) | Vijayakumar et al. (2006) | Wolmer et al. (2005) | Wolmer et al. (2003) |  |
| Screening questions (for all types) | S1. Are there clear research questions? | 1 | 1 | 1 | 1 | 1 | 1 | 1 | 1 | 1 | 1 | 1 | 1 | 1 | 1 | 1 | 1 | 1 | 1 |  |
|  | S2. Do the collected data allow to address the research questions? | 1 | 1 | 1 | 1 | 1 | 1 | 1 | 1 | 1 | 1 | 1 | 1 | 1 | 1 | 1 | 1 | 1 | 1 |  |
|  | *Further appraisal may not be feasible or appropriate when the answer is ‘No’ or ‘Can’t tell’ to one or both screening questions.* | | | | | | | |  | | | | |  | | | | | |  |
| 1. Quantitative randomized controlled trials | 1.1. Is randomization appropriately performed? | 1 | n/a | 1 | n/a | 1 | n/a | n/a | 1 | 1 | n/a | 1 | 1 | n/a | 1 | n/a | 1 | 1 | n/a |  |
|  | 1.2. Are the groups comparable at baseline? | 1 | n/a | 1 | n/a | 1 | n/a | n/a | 1 | 1 | n/a | 1 | 0.5 | n/a | 1 | n/a | 1 | 1 | n/a |  |
|  | 1.3. Are there complete outcome data? | 1 | n/a | 1 | n/a | 1 | n/a | n/a | 1 | 1 | n/a | 1 | 1 | n/a | 1 | n/a | 1 | 1 | n/a |  |
|  | 1.4. Are outcome assessors blinded to the intervention provided? | 0.5 | n/a | 1 | n/a | 0.5 | n/a | n/a | 0 | 0 | n/a | 0.5 | 0 | n/a | 1 | n/a | 0.5 | 0.5 | n/a |  |
|  | 1.5. Did the participants adhere to the assigned intervention? | 1 | n/a | 1 | n/a | 1 | n/a | n/a | 1 | 1 | n/a | 0 | 0.5 | n/a | 1 | n/a | 0 | 0.5 | n/a |  |
| 2. Quantitative pretest-posttest Design | 2.1. Are the participants representative of the target population? | n/a | 1 | n/a | 1 | n/a | 1 | 1 | n/a | n/a | 1 | n/a | n/a | 1 | n/a | 1 | n/a | n/a | 1 | |
|  | 2.2. Are measurements appropriate regarding both the outcome and intervention (or exposure)? | n/a | 1 | n/a | 1 | n/a | 1 | 1 | n/a | n/a | 1 | n/a | n/a | 1 | n/a | 1 | n/a | n/a | 1 | |
|  | 2.3. Are there complete outcome data? | n/a | 1 | n/a | 1 | n/a | 1 | 1 | n/a | n/a | 1 | n/a | n/a | 1 | n/a | 1 | n/a | n/a | 0.5 | |
|  | 2.4. Are the confounders accounted for in the design and analysis? | n/a | 0.5 | n/a | 1 | n/a | 0.5 | 0.5 | n/a | n/a | 1 | n/a | n/a | 0.5 | n/a | 0.5 | n/a | n/a | 1 |  |
|  | 2.5 During the study period, is the intervention administered (or exposure occurred) as intended? | n/a | 1 | n/a | 1 | n/a | 1 | 1 | n/a | n/a | 1 | n/a | n/a | 1 | n/a | 1 | n/a | n/a | 1 |  |
| Total Score |  | 6.5 | 6.5 | 7 | 7 | 6.5 | 6.5 | 6.5 | 6.5 | 6 | 7 | 5.5 | 5 | 6.5 | 7 | 6.5 | 5.5 | 6 | 6.5 |  |

Appendix 3. Data Extraction of Studies

| Author (Year) | Study Design | Settings | | | | | | Measurements | Interventions | | | outcomes |
| --- | --- | --- | --- | --- | --- | --- | --- | --- | --- | --- | --- | --- |
|  |  | Countries | Disaster | Populations  (Age & N) | | Places | Start time of intervention |  | Types | Forms | Qualification of therapists |  |
| Berger & Gelkopf (2009) | Quantitative Quasi-RCT | Welligama, Sri Lanka | Tsunami | 9-14y | 166 | School | 14 months | UPID | ERASE Stress program | Group sessions by face to face. Each session included 12-16 students. | Homeroom teachers | The PTSD scores in the ES-SL group showed significant improvement over time (*p* < .001).  The probable PTSD diagnosis rate decreased dramatically (33.3% → 6.0%) in the ES-SL group, while the WL group showed almost no change (31.7% → 28.1%), even with 3 new PTSD diagnoses. The chi-square test indicated a significant difference in the PTSD change trends between the two groups (χ² = 14.02, *p* = .001) .  A covariance analysis showed a distinct time × group × baseline interaction for PTSD severity (*p* < .001).  Pearson correlation showed that students with higher baseline PTSD scores had greater improvement (r = .70).  The improvement in symptoms was greater in students with probably PTSD compared to those without PTSD (*p* = .006). |
| Cain et al. (2010) | Quantitative pretest-posttest Design | Louisiana State, USA | Hurricane | 5-15y, M = 9.5 | 99 | School/FEMA trailer park | 20 months | CPTSD-RI | PFA |  | licensed clinical social workers &  graduate level social work student assistants | Children showed a statistically significant reduction in PTSD symptoms (*t* = 2.25, *p* = 0.027), with a mean score of 30.22. This finding indicated that although PTSD symptoms improved significantly, the average symptom level remained within the moderate range. |
| Catani et al. (2009) | Quantitative RCT | Vadamarachchi, Sri Lanka | Tsunami | 8-14y | 31 | Refugee camp | 3 weeks | UPID | NET & MED-RELAX | Face-to-face. | Teachers | Treatments had a main time effect on UPID total score (*p* < .001).  Compared with pretest, effect sizes of the NET group were 1.76, CI [0.9-2.5] at posttest and 1.96, CI [1.1-2.8] at 6-month follow-up, and the MED-RELAX group were 1.83, CI [0.9-2.6] and 2.20, CI [1.2-3.0].  No significant difference between the effects of the two treatment modalities was observed across time points.  Six months after treatment, respectively in the NET and MED-RELAX groups, 81% and 71% of children did not meet the PTSD-RI threshold for a PTSD diagnosis. |

| Author (Year) | Study Design | Settings | | | | | | Measurements | Interventions | | | outcomes |
| --- | --- | --- | --- | --- | --- | --- | --- | --- | --- | --- | --- | --- |
|  |  | Countries | Disaster | Populations  (Age & N) | | Places | Start time of intervention |  | Types | Forms | Qualification of therapists |  |
| Cobham & McDermott (2024) | Quantitative pretest-posttest Design | Queensland, Australia | Floods | 3-7 grades, M = 9.85 | 19 | School | 5 months | PTSD-RI | TF-CBT | Individual and parents conjoint intervention. | Clinicians | From pre-treatment to 12-month follow-up, the mean total PTSD-RI symptom score decreased significantly from 47.95 to 7.00, 95% CI [0.52-0.80].  Seventeen children reached criteria for a full PTSD diagnosis and two for a sub-clinical diagnosis at pre-treatment, while no children met criteria for either at 12-month follow-up. |
| Dhital et al. (2019) | Quantitative RCT | Dhading, Nepal | Earthquake | 6-8 grades, M = 13 | 1220 | School | 17 months | CPSS | Psycho-social support | Each school as a group by face-to-face. | Teachers | At baseline, the PTSD scores in the intervention group were slightly lower than those in the control group, with a statistically significant difference (*p* = .025). However, after the intervention, there was no significant difference in the mean PTSD scores between the two groups (*p* = .477).  Further GEE analysis indicated that the intervention had no significant effect on PTSD scores over time (*p* = .536), nor was there a significant difference between the intervention and control schools (*p* = .343). |
| Fernandez (2007) | Quantitative pretest-posttest Design | Molise, Italy | Earthquake | 7-11y | 22 | School | 1 month | A directly administered questionnaire prepared by the National Institute of  Health which consists of a list of typical PTSD symptoms. | EMDR | Individual sessions by face-to-face.  Parents were allowed to attend children’s sessions. | Members of mental  health public agencies (ASL) and the EMDR  Association | The overall PTSD symptoms were significantly reduced at the 0.01 level (*p* = .003). Each symptom cluster was slightly below the 0.01 significance level, but they were all significant at the 0.05 level (Avoidance *p* = .015, Intrusiveness *p* = .011, Arousal *p* = .015).  During the acute phase following the trauma, the proportion of the sample meeting PTSD criteria remained at a similar level, with no significant decrease after the first treatment cycle. However, the proportion gradually decreased thereafter, from an initial 61% to a final 9%. |
| Giannopoulou (2006) | Quantitative pretest-posttest Design | Athens, Greece | Earthquake | 8-12y, M = 9.6 | 17 | Community | 2 months (group 1)  4 months (group 2) | CRIES-13 | CBT | Group sessions by face-to-face. | Child and adolescent  psychiatrists experienced in CBT & clinical psychologists trained in treating PTSD | ANOVA indicated significant changes in PTSD across different time points (*p* < .001).  Pairwise comparisons showed a significant decrease in CRIES-13 scores after treatment (*p* < .001), a further reduction at the 18-month follow-up (*p* < .001), but no significant change at the 4-year follow-up (*p* > .05). |

| Author (Year) | Study Design | Settings | | | | | | Measurements |  | Interventions |  | outcomes |
| --- | --- | --- | --- | --- | --- | --- | --- | --- | --- | --- | --- | --- |
|  |  | Countries | Disaster | Populations  (Age & N) | | Places | Start time of intervention |  | Types | Forms | Qualification of therapists |  |
| Goenjian et al. (2021)  [25-year follow-up of Goenjian et al. (2005)] | Quantitative RCT | Gumri,  Armenia | Earthquake | M(treated) = 36.18,  M(not-treated) = 38.24 | 75 | N/A | 25-year long-term follow-up | PTSD-RI & PCL | N/A | N/A | N/A | PTSD-RI scores of the treated students were significantly lower than students in the control group (*p* < .05).  The mean change of the treated group was significantly greater than that of the control group (*p* < .03).  PCL scores of the treated group were less than the not-treated group (*p* < .02). |
| Goenjian et al. (2005) | Quantitative RCT | Gumri,  Armenia | Earthquake | 15-17y | 63 | School | 1.5 years | CPTSD-RI | TF-CBT & psycho-education | Both individual and group interventions were conducted, by face-to-face. | Psychiatric outreach program staff | A significant decrease in CPTSD-RI scores was observed in students 3.5 years after the treatment ended (*p* < .001), and the mean change was 3 times greater than that of the students not treated.  Scores of the treated students were significantly lower than those in the control group (*p* < .01). |
| Graham et al. (2017) | Quantitative pretest-posttest Design | Louisiana State, USA | Hurricane | 8-17y,  M = 12.16 | 112 | School | 4 months | TSCC | CBITS & TF-CBT | Individual sessions by face-to-face. | Clinicians | TSCC showed a significant decrease on total score (*p* < .001).  Significant reduction was found in overall TSCC total scale from baseline to Follow-up 1 (*p* < .001) and to Follow-up 2 (*p* < .001), but not from Follow-up 1 to Follow-up 2. |
| Jaycox et al. (2010) | Quantitative RCT | Louisiana State, USA | Hurricane | 4-8 grades, M = 11.6 | 118 | Clinics/  school | 15 Months | UPID & CPSS | CBITS & TF-CBT | CBITS included individual and group interventions, TF-CBT included individual and parents conjoint intervention. | N/A | Ten months after the treatment, the mean PTSD scores of the TF-CBT group shifted to the normal range, with those in the CBITS group fell into the lower clinical range.  The proportion of children with PTSD symptom scores in the risk range (CPSS score ≥ 12) was 65% in the CBITS group and 43% in the TF-CBT group, which were comparable (*p* = .22).  Of the nine children who completed the whole TF-CBT treatment, only one still met the diagnostic criteria for PTSD. |
| Osofsky et al. (2018) | Quantitative RCT | Louisiana State, USA | Hurricane | 15-17y, M = 15.7 | 212 | School/  community | 9 months | SQS & UPID | The YLP | Group sessions by face-to-face. | Teachers & mental health professionals | There were decreases in trauma symptoms in students in the YLP, and this change was significantly associated with the increase of their self-efficacy (*p* < .001).  The raw data of how much the trauma symptoms changed was not provided.  No change of trauma symptoms was observed in the non-YLP group as their self-efficacy was not improved. |
| Plummer et al. (2008) | Quantitative pretest-posttest Design | Louisiana State, USA | Hurricane | 6-15y | 12 | School | 6 months | The Reaction Index (a questionnaire  based on CPTSD-RI) | PFA | Group sessions by face-to-face. | Licensed clinical social workers | There were no statistically significant differences in PTSD symptoms, *t* = .32, *p* = .756; however, a slight reduction in mean symptom scores was observed (from M = 28.4 to M = 27.4). This indicates that, on average, children still experienced moderate levels of PTSD symptoms. |

| Author (Year) | Study Design | Settings | | | | | | Measurements | Interventions | | | outcomes |
| --- | --- | --- | --- | --- | --- | --- | --- | --- | --- | --- | --- | --- |
|  |  | Countries | Disaster | Populations  (Age & N) | | Places | Start time of intervention |  | Types | Forms | Qualification of therapists |  |
| Salloum & Overstreet (2008) | Quantitative RCT | Louisiana State, USA | Hurricane | 2-6 grades, 7-12y | 56 | School/  home | 4 months | UPID | Project LAST (TF-CBT & NET) | Both individual and group interventions were conducted, by face-to-face. Each group had 5 to 6 children. | Clinicians | A significant main effect of time in PTSD symptoms was found (*p* < .001).  Significant decreases were indicated by post hoc analyses from pre-treatment to post-treatment (*p* = .001) and to follow-up (*p* = .001).  53% of the children were classified within the clinical range for PTSD at pre-treatment, which decreased to 13% at post-treatment, and to 4% at follow-up.  The age × gender interaction for PTSD symptoms also approached significance (*p* = .054, partial η² = .082), with younger girls (7–9 years; M = 36.7) showing the least improvement compared to younger boys (7–9 years; M = 30.1), older girls (10–12 years; M = 23.3), and older boys (10–12 years; M = 29.7). |
| Trentini et al. (2018) | Quantitative pretest-posttest Design | Umbria, Italy | Earthquake | 5-13y, M = 9.15 | 332 | N/A | 1 day | CRIES-13 | EMDR-IGTP | Group sessions online. Each group has 7-24 children. | EMDR therapists | The results from the CRIES-13 scale showed that the overall effect of EMDR-IGTP was significant (Cohen’s f² = 1.19), with no significant differences between groups.  Earlier treatment led to greater symptom improvement (F = 17.331, *p* = .001, η²_p_ = .12).  Older children benefited more (F = 72.186, *p* = .001, η²_p_ = .36).  Girls showed greater symptom improvement than boys (F = 5.693, *p* = .004, η²_p_ = .04). |
| Vijayakumar et al. (2006) | Quantitative RCT | Chennai,  India | Tsunami | 11-14y | 135 | Community | 11 months | CPTSD-RI | Play therapy | Group sessions by face-to-face. | Three volunteers & Two psychologists | Although PTSD symptoms decreased after treatment, it was not significant (*p* = .964), while the comparable control group was not found to differ before and after the treatment. |
| Wolmer et al. (2005)  [3-year follow-up of Wolmer et al. (2003)] | Quantitative RCT | Adapazari, Turkey | Earthquake | 9-17y, M = 11.5 | 287 | N/A | 3-year follow-up | CPTSD-RI & TDGS | N/A | N/A | N/A | Nearly 18% of the children still reported severe PTSD symptoms, and significant decrease was observed in all three domains: posttraumatic stress (*p* < .02), dissociation (*p* < .001) and grief (*p* < .001).  29% of the children experienced an increase in PTSS severity, 30% remained at the same level, and 41% moved to a lower severity category.  No significant difference in PTSD symptoms were found between the children that received treatment and those in the control group with the same exposure. |

| Author (Year) | Study Design | Settings | | | | | | Measurements | Interventions | | | outcomes |
| --- | --- | --- | --- | --- | --- | --- | --- | --- | --- | --- | --- | --- |
|  |  | Countries | Disaster | Populations  (Age & N) | | Places | Start time of intervention |  | Types | Forms | Qualification of therapists |  |
| Wolmer et al. (2003) | Quantitative pretest-posttest Design | Adapazari, Turkey | Earthquake | 1-5 grades, M = 8.2 | 202 | School | 4 months | TDGS & Turkish version of CPTSD-RI | CBT & psycho-education | Each class as a group by face-to-face. | Teachers | The mean severity of posttraumatic stress (*p* < .001) and traumatic dissociation (*p* < .001) decreased significantly, while grief (*p* < .001) increased.  The percentage of children with severe and very severe PTSS dropped from 30% to 18%, which was similar to the proportion of children not directly affected by the earthquake.  27.5% of the children experienced an increase in PTSS severity, 33.5% remained at the same level, and 39% moved to a lower severity category.  41% of the children whose PTSS level increased to the “severe” category were in the same school year. |

*Note.* N/A = Not Available, M = Mean, N = Number of Population;

CBITS = Cognitive Behavioral Intervention for Trauma in Schools, CBT = Cognitive Behavioral Therapy, CPSS = Child PTSD Symptom Scale, CPTSD-RI = Child Post-Traumatic Stress Disorder Reaction Index, CRIES-13 = Children’s Revised Impact of Event Scale, EMDR = Eye Movement Desensitization and Reprocessing, EMDR-IGTP = EMDR Integrative Group Treatment Protocol, KRI = Kauai Recovery Inventory, MED-RELAX = meditation-relaxation, NET = Narrative Exposure Therapy, PCL = PTSD Check List, PFA = Psychological First Aid, SQS = Status Questionnaire for Students, TDGS = Traumatic Dissociation and Grief Scale, TF-CBT = Trauma-focused CBT, TSCC = Trauma Symptom Checklist for Children, UPID = UCLA PTSD Index for DSM-IV, YLP = Young Leadership Program ;

*The exact number of population is not provided.
